# Dose-dependent functions of fibroblast growth factor 9 regulate the fate of murine XY primordial germ cells^†^

**DOI:** 10.1095/biolreprod.116.143941

**Published:** 2016-12-23

**Authors:** Ferhat Ulu, Sung-Min Kim, Toshifumi Yokoyama, Yukiko Yamazaki

**Affiliations:** 1 Institute for Biogenesis Research, John A. Burns School of Medicine, University of Hawaii, Honolulu, Hawaii, USA; 2 Department of Animal Science, Kobe University, Kobe, Hyogo, Japan

**Keywords:** FGF9, XY primordial germ cells, male differentiation, cell proliferation, p38 signaling pathway, ERK1/2 signaling pathway

## Abstract

Male differentiation of primordial germ cells (PGCs) is initiated by the inhibition of entry into meiosis and exposure to male-inducing factor(s), which are regulated by somatic elements of the developing gonad. Fibroblast growth factor 9 (FGF9) produced by pre-Sertoli cells is essential for male gonadal differentiation and also contributes to survival and male differentiation of XY PGCs. However, it is not clear how FGF9 regulates PGC fate. Using a PGC culture system, we identified dose-dependent, fate-determining functions of FGF9 in XY PGCs. Treatment with low levels of FGF9 (0.2 ng/ml) increased expression of male-specific *Dnmt3L* and *Nanos2* in XY PGCs. Conversely, treatment with high levels of FGF9 (25 ng/ml) suppressed male-specific gene expression and stimulated proliferation of XY PGCs. Western blotting showed that low FGF9 treatment enhanced p38 MAPK (mitogen-activated protein kinase) phosphorylation in the same cells. In contrast, high FGF9 treatment significantly stimulated the ERK (extracellular signal-regulated kinase)1/2 signaling pathway in XY PGCs. We investigated the relationship between the ERK1/2 signaling pathway stimulated by high FGF9 and regulation of PGC proliferation. An ERK1/2 inhibitor (U0126) suppressed the PGC proliferation that would otherwise be stimulated by high FGF9 treatment, and increased *Nanos2* expression in XY PGCs. Conversely, a p38 MAPK inhibitor (SB202190) significantly suppressed *Nanos2* expression that would otherwise be stimulated by low FGF9 in XY PGCs. Taken together, our results suggest that stage-specific expression of FGF9 in XY gonads regulates the balance between proliferation and differentiation of XY PGCs in a dose-dependent manner.

## Summary Sentence

FGF9 directly regulates different fates of XY PGCs in a dose-dependent manner: low FGF9 treatment promotes p38 signaling pathway to induce PGC male differentiation, whereas high FGF9 treatment enhances ERK1/2 signaling pathway to proliferate PGCs.

## Introduction

The sex-specific phenotypic fate of mammalian primordial germ cells (PGCs) is induced extrinsically by the gonadal environment rather than intrinsically by sex chromosome constitution (XX, XY) [1]. PGCs initiate differentiation as either prospermatogonia or oogonia, depending on the sexual phenotype of the surrounding gonadal somatic cells [2]. In mice, PGCs initiate sex differentiation after migrating to the genital ridge at around 10.5 days postcoitum (dpc). In female gonads, wingless-related MMTV integration site (Wnt) signaling and retinoic acid (RA) are essential for oogenesis. Wnt signaling promotes female differentiation and suppresses male differentiation pathway [3–5]. Retinoic acid signaling plays an essential role in meiotic initiation in female gonads. Retinoic acid induces the mRNA expression of stimulated by retinoic acid gene 8 (*Stra8*), an RA responsive gene, which then leads to meiotic initiation in XX PGCs at 12.5 dpc [6–9]. In male gonads, expression of sex determining region Y (*Sry*) occurs in Sertoli cell precursors to upregulate *Sox9* (*Sry-*box containing gene 9) and fibroblast growth factor 9 (*Fgf9*) to promote Sertoli cell differentiation and testis development [10–14].

*Fgf9* is initially expressed in gonads of both sexes, but then shows upregulated expression uniquely in the XY gonad shortly after initiation of SRY and SOX9 expression [15–17]. Between 11.0–11.5 dpc, FGF9 promotes proliferation and differentiation of pre-Sertoli cells to induce development of the testis [18]. At 12.5–14.5 dpc, *Fgf9* expression gradually decreases while testis differentiation progresses [17]. Deletion of the *Fgf9* gene leads to male-to-female sex reversal in XY fetuses, suggesting that FGF9 is essential for normal male sex determination [11,15,18]. FGF9 is also crucial for survival of XY PGCs, suggesting a sex-specific role for FGF9 in germ cells and in gonadal somatic cells [19]. In 2010, it was reported that FGF9 and RA act antagonistically to determine germ cell fates [17,20]. FGF9 upregulates *Nanos2* mRNA levels in female and male germ cells and inhibits meiosis in both sexes [20]. Bowles et al. proposed that FGF9 acts directly on PGCs to inhibit *Stra8* upregulation and induce expression of the male-specific marker *Nanos2* [17].

Recently, it was found that Nodal/Activin signaling contributes to male germ cell differentiation [21–24]. It was also reported that p38 MAPK (mitogen-activated protein kinase) signaling is activated in XY PGCs, but not in XX PGCs at about the time that the mouse gonads initiate sex differentiation [25]. When XY gonads at 11.5 dpc were cultured with p38 inhibitors, POU5F1 expression was downregulated and *Stra8* and SYCP3 were upregulated, suggesting that p38 MARK signaling antagonizes entry of XY PGCs into meiosis, and, instead, direct PGCs toward mitotic quiescence and male differentiation [25]. Wu et al. further demonstrated that two intrinsic signals, SMAD2 and p38, act in XY PGCs to induce *Nanos2* expression and to suppress meiosis, respectively [23]. They proposed a model in which SMAD2 and p38 signaling coordinate to determine the sexual fate of XY PGCs, although it is still unknown how these signaling pathways are initiated in the developing testis [23]. A previous study suggested that FGF9 treatment induces expression of CRIPTO, a cofactor of Nodal, implying that FGF9 triggers Nodal signaling in XY PGCs [26]. However, treatment of PGCs with FGF9 did not enhance *Nodal* expression, suggesting *Nodal* expression is not regulated by FGF9 in these cells. In contrast, XY PGCs in fetal testes lacking *Fgf9* and *Wnt4* enter the male pathway normally, indicating the primary role of FGF9 signaling is the repression of female-promoting genes, and excluding the possibility that FGF9 is directly required for Nodal/Activin activation and male differentiation [23,27]. However, it remains unclear exactly how FGF9 stimulates downstream targets of signal transduction pathways to regulate the sex-specific fate of PGCs.

In this study, we have used our PGC culture system to investigate the direct effect of FGF9 on determination of XY PGC fate [8,28,29]. We first examined dose-dependent effects of FGF9 on XY PGC differentiation. This has allowed us to identify PGC-specific signaling transduction pathways that are promoted by dose-dependent FGF9 treatment and regulate the sex-specific fate of XY PGC differentiation in vitro.

## Materials and methods

### Mice

Mice carrying a *Pou5f1*-green fluorescent protein (GFP) transgene (Tg OG2) were generated by microinjecting (CBA/Caj X C57BL/6J) F_2_ zygotes and express germ cell-specific GFP driven by the *pou5fl* gene promoter/enhancer (a generous gift from Dr. Jeff R. Mann, University of Melbourne, Melbourne, VIC, Australia) [30]. Female CD-1 mice (Charles River) were mated with Tg OG2 homozygous males to produce (CD-1 X OG2) F_1_ hybrid fetuses. GFP-positive PGCs sorted from these F1 fetuses were subjected to all experiments. All experiments involving mice were reviewed and approved in advance by the Institutional Animal Care and Use Committee of the University of Hawaii, and all procedures were in accordance with guidelines recommended by the NIH.

### Media

Gonads were dissected in Hepes-Dulbecco Modified Eagle Medium (DMEM; Invitrogen, Carlsbad, CA) supplemented with 15% fetal bovine serum (FBS; Hyclone Laboratories, Logan, UT). PGCs were cultured in high-glucose DMEM supplemented with 0.1 mM nonessential amino acids, 0.1 mM 2-merkaptoethanol, 100 IU/ml penicillin, 100 μg/ml streptomycin, 2 mM glutamine, 1 mM sodium pyruvate, and 15% FBS. Isolated PGCs were treated with 0.2–100 ng/ml FGF9 (Sigma-Aldrich, St. Louis, MO). In some experiments, PGCs were cultured with p38 inhibitors (BIRB796; Cayman Chemical Company, Ann Arbor, MI, SB202190; Sigma-Aldrich) at 10 μM or MEK inhibitor (U0126; Cayman Chemical Company) at 1 or 10 μM.

### XY genotyping

Fetuses were sexed based on testis cord formation at 12.5 dpc. Sexing of 11.5 dpc fetuses was accomplished by PCR using sex-determining region of Y chromosome (*Sry*) specific primers (5^΄^-CTGTGTAGGATCTTCAATCTCT-3^΄^ and 5^΄^-GTGGTGAGAGGCACAAGTTGGC-3^΄^). Heads of the fetuses were individually collected to boil for 10 min, then centrifuged at 13,200 rpm at 4^°^C for 5 min, and the supernatant was then subjected to PCR amplification analysis.

### Primordial germ cell isolation and culture

XY and XX PGCs were isolated from the gonads for in vitro culture as previously described [8,28,29]. Briefly, male and female gonads obtained from (CD-1 X OG2) F_1_ fetuses at 11.5 and 12.5 dpc were dissected in Hepes- DMEM supplemented with 15% FBS and incubated in 0.2% Collagenase (Calbiochem, San Diego, CA) and Accumax (Innovative Cell Technologies, San Diego) for 10 min at 37^°^C, respectively. After enzymatic treatment, gonads were mechanically dissociated and filtered through a 40 μm cell strainer (BD Falcon, Franklin Lakes, NJ) to prepare a single cell suspension. GFP-positive PGCs were sorted using a FACS Aria system (BD Bioscience, San Jose, CA). The purity of GFP-positive cells was >95%. About 8,000 PGCs were cultured on collagen-coated mesh inserts (Corning Life Science, Lowell, MA) for 1–3 days.

### Quantitative gene expression analysis

After 1–3 days of culture, morphologically healthy PGCs were manually selected with a glass pipette using an inverted microscope (X200) (Olympus IX71, Center Valley, PA). About 200–800 PGCs were collected as one set, and cDNA was synthesized using the SuperScript III Cells Direct cDNA synthesis kit (Invitrogen). Quantitative PCR analysis was performed using the MyIQ Single-Color Real-Time PCR Detection system (Bio-Rad Laboratories, Hercules, CA). Three to five experiments were repeated independently for each culture condition. As previously shown [29], the sequences of primers used were (forward and reverse, respectively) as follows: for DNA methyltransferase 3-like (*Dnmt3L)*, 5^΄^-GTGCGGGTACTGAGCCTTTTTAGA-3^΄^ and 5^΄^-CGACATTTGTGACATCTTCCACGTA-3^΄^; for *Stra8*, 5^΄^-GTTTCCTGCGTGTTCCACAAG-3^΄^ and 5^΄^-CACCCGAGGCTCAAGCTTC-3^΄^; for β-actin, 5^΄^-CCTGTATGCCTCTGGTCGTA-3^΄^ and 5^΄^-CCATCTCCTGCTCGAAGTCT-3^΄^. The primer set for *Nanos2* (Mm02525720_s1) was obtained from Applied Biosystems (Waltham, MA). The results were normalized to levels detected for *β-actin* gene expression and the levels of transcripts were presented as relative expression to control groups which were arbitrarily set as 1.00 as previously shown [8,28,29].

### Cell proliferation analysis

Cell proliferation was analyzed using the Click-IT EdU (5-ethynyl-2^΄^-deoxyuridine) Alexa Flour HCS assay kit (Invitrogen). After 2 days of culture, PGCs were incubated with EdU for 5 h at 37^°^C. After incubation, the cells were picked up randomly, placed on glass slides, and treated according to the manufacturer's instructions. EdU-positive cells were counted using an Axio Scope A1 microscope (Carl Zeiss Microimaging, Göttingen, Germany). We repeated experiments at least three times and analyzed a total of 200–300 germ cells for each culture condition. The % of EdU-positive cells was calculated per cells analyzed.

### Protein preparation and western blotting

For western blot analysis, cell lysates were prepared from 10,000 PGCs (in some case 5,000 PGCs) incubated for 30 min - 2 h under different conditions. The equal amounts of cell lysates were loaded into each lane of a 12% SDS-PAGE gel and electrotransferred onto polyvinyl difluoride (PVDF) membranes (GE Healthcare, Chicago, IL). The transferred membranes were blocked with 5% nonfat dried milk in 0.1% Tween 20 in PBS (PBS-T). The membranes were probed with primary antibodies (all purchased from Cell Signaling Technology, Danvers, MA) against β-ACTIN (1:1000), ERK (extracellular signal-regulated kinase)1/2 (1:1000), phospho-ERK1/2 (1:1000), p38 (1:500), phospho-p38 (1:500), AKT (1:500), phospho-AKT (1:500), JNK (c-Jun N-terminal kinase) (1:500), and phospho-JNK (1:500) overnight at 4^°^C. The following day, the membranes were rinsed three times with PBS-T for 10 min each and incubated with an anti-rabbit IgG, HRP-linked secondary antibody (1:1000, Cell Signaling Technology) for 1 h at room temperature. Information of antibodies used for western blotting is summarized in Supplemental Table S1. The membranes were exposed on X-ray film (GE Healthcare) to visualize the protein bands. The protein bands were quantified by densitometry using Image J software (NIH, Bethesda, MD) and normalized to the β-ACTIN levels, respectively. The relative optical densities of phosphorylated protein levels were calculated based on the control levels set as 1.00. In this study, the western blotting analysis was repeated twice in each experiment.

### Statistical analysis

Data are represented as the mean ± standard errors of the means (SEM). For comparison of quantitative mRNA expression levels and cell proliferation ratios, data were analyzed by one-way analysis of variance followed by the Tukey's multiple comparison test for normally distributed data, or the Kruskal-Wallis test with the Mann-Whitney post hoc test. A *P*-value < 0.05 was considered statistically significant. All statistical analyses were performed using Graphpad Prism 4.0 (Graphpad Software Inc., San Diego).

## Results

### Dose-dependent effect of fibroblast growth factor 9 on male differentiation of primordial germ cells

**Figure 1.**
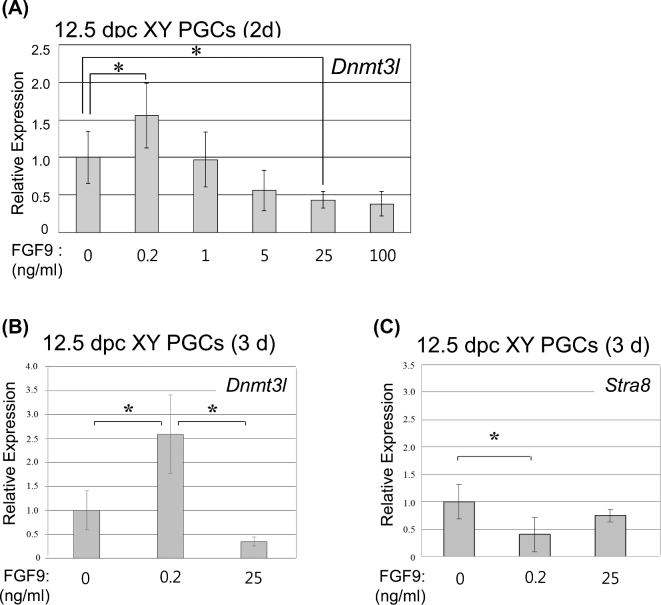
Quantitative expression of *Dnmt3l* in XY PGCs cultured with FGF9 at different concentrations. **A)** XY PGCs isolated at 12.5 dpc were cultured with FGF9 at various concentrations (0, 0.2, 1, 5, 25 and 100 ng/ml) for 2 days. After culture, the cells were collected manually and subjected to cDNA synthesis. In the cells treated with FGF9, *Dnmt3l* mRNA expression reached a peak at 0.2 ng/ml (*P* < 0.05), then gradually decreased in a dose-dependent manner. At 25 (*P* < 0.05) or 100 ng/ml FGF9 suppressed *Dnmt3l* expression in XY PGCs. Based on these mRNA expression levels of *Dnmt3l*, 0.2 ng/ml FGF9 was designated as low FGF, and 25 ng/ml FGF9 was designated as high FGF9, respectively, for all subsequent experiments. **B, C)** XY PGCs isolated at 12.5 dpc were cultured with low (0.2 ng/ml) or high (25ng/ml) FGF9 for 3 days to examine *Dnmt3l* (B) or *Stra8* (C) mRNA expression levels. Low FGF9 treatment promoted *Dnmt3l* (*P* < 0.05), but suppressed *Stra8* mRNA expression (*P* < 0.05). In contrast, high FGF9 significantly suppressed *Dnmt3l* mRNA expression (*P* < 0.05), but did not promote S*tra8* expression in the cells. Results were normalized to the β-actin mRNA expression. All expression values were calculated relative to control levels set at 1.0. Data represent the mean ±SEM. ^*^P < 0.05.

In the male gonad, PGCs initiate male differentiation at around 12.5 dpc [29]. To clarify direct effects of FGF9 on male differentiation of PGCs, 12.5 dpc XY PGCs were cultured with different concentrations of FGF9 (0, 0.2, 1, 5, 25, and 100 ng/ml) for 2 days and male-specific *Dnmt3l* mRNA expression was examined (Figure 1A). In the presence of 0.2 ng/ml FGF9, *Dnmt3l* expression peaked at 1.5-fold higher than the control (*P* < 0.05). Then, the mRNA levels were gradually decreased under FGF9 concentrations at 1–100 ng/ml. In particular, in the presence of 25 ng/ml FGF9 *Dnmt3l* expression was significantly suppressed in XY PGCs to a level less than half that seen in the control (*P* < 0.05) (Figure 1A). To better define the dose-dependent effects of FGF9 concentration on PGC fate, we examined two conditions—a low (0.2 ng/ml) and a high (25 ng/ml) concentration of FGF9 supplementation in the media for the experiments described below.

XY PGCs were recovered from fetuses at 12.5 dpc and cultured with the low or high concentrations of FGF9 for 3 days, and *Dnmt3l* mRNA levels were assessed during this period (Figure 1B). Low FGF9 treatment increased *Dnmt3l* mRNA level in XY PGCs to a level more than 2.5-fold higher than the control (*P* < 0.05). In contrast, high FGF9 treatment significantly suppressed *Dnmt3l* expression to a level less than half of the control (*P* < 0.05) (Figure 1B). To identify whether dose-dependent FGF9 treatments affect PGC female differentiation, we analyzed the mRNA level of *Stra8* which induces meiotic initiation of XX PGCs in fetal ovaries (Figure 1C). XY PGCs cultured with low FGF9 decreased *Stra8* mRNA expression to less than half of the control (*P* < 0.05). The cells cultured with high FGF9 also showed lower *Stra8* mRNA level compared with the control (Figure 1C). These results suggest that low FGF9 treatment induces 12.5 dpc XY PGCs to enter the male pathway, whereas high FGF9 treatment prevents XY PGCs from entering either the male or female pathway.

**Figure 2.**
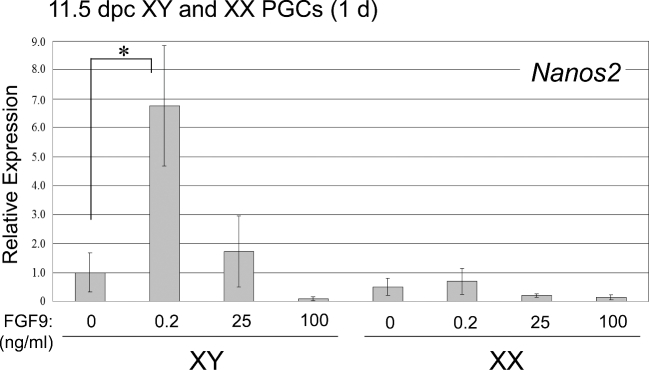
Quantitative expression of *Nanos2* in XY and XX PGCs cultured with FGF9 at different concentrations. Isolated XY and XX PGCs at 11.5 dpc were cultured with FGF9 at various concentrations (0, 0.2, 25 and 100 ng/ml) for 1 day to be subjected to cDNA synthesis. In XY PGCs, expression of *Nanos2* mRNA dramatically increased following low FGF9 (0.2 ng/ml) treatment (*P* < 0.05), then decreased in a dose-dependent manner (*P* > 0.05). In XX PGCs, *Nanos2* mRNA expression was not promoted regardless of the FGF9 concentration. Results were normalized to the β-actin mRNA expression. All expression values were calculated relative to control levels set at 1.0. Data represent the mean ±SEM. ^*^P < 0.05.

XY and XX PGCs at 11.5 dpc are not sexually differentiated yet. We examined whether low or high FGF9 treatment induces male differentiation of indifferent PGCs regardless of their sex chromosome constitution. We cultured both XY and XX PGCs from 11.5 dpc embryos in the presence of different concentrations of FGF9 (0, 0.2, 25, 100 ng/ml) for 1 day and analyzed mRNA levels of male-specific *Nanos 2* (Figure 2). XY PGCs cultured in the presence of 0.2 ng/ml (low FGF9) showed a significant increase in expression of *Nanos2* mRNA to a level equal to approximately 7-fold higher than the control (*P* < 0.05), while the presence of higher levels of FGF9 resulted in decreased mRNA expression of *Nanos2* in a dose-dependent manner (25 and 100 ng/ml) (*P* > 0.05). In contrast, XX PGCs cultured in the presence of different levels of FGF9 showed no significant increase in *Nanos2* mRNA levels regardless of the concentration (*P* > 0.05) (Figure 2). These data strongly suggest that low levels of FGF9 promote male differentiation of PGCs in an intrinsic, sex-specific manner such that only XY PGCs respond to this stimulation by upregulating *Nanos2* expression.

### Primordial germ cell proliferation under low fibroblast growth factor 9 treatment

**Figure 3.**
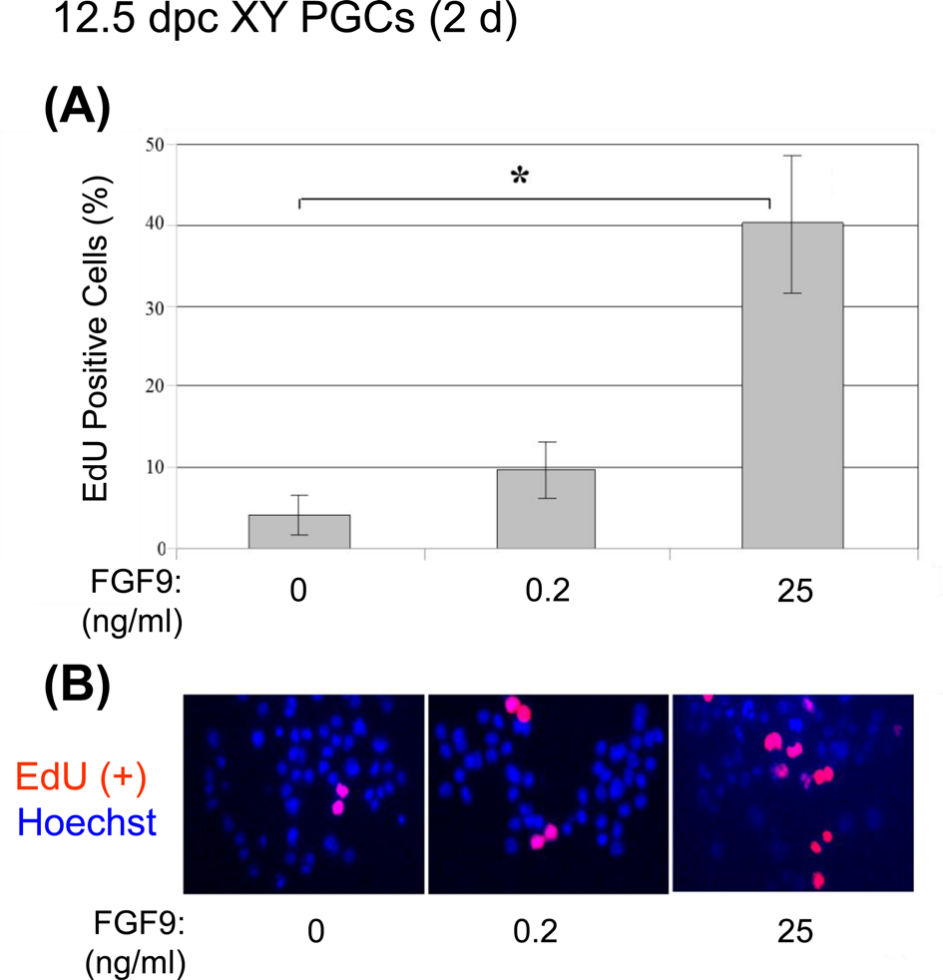
Proliferation of XY PGCs cultured with low and high FGF9. XY PGCs isolated at 12.5 dpc and cultured with low or high FGF9 for 2 days were subjected to an EdU-based cell proliferation assay. After culture, the cells were incubated with EdU for 5 hr and EdU-positive cells were counted (A). EdU-positive cells were detected as red fluorescent cells among Hoechst-positive cells under a fluorescence microscope (original magnification x400) (B). The ratio of EdU positive-cells was significantly increased under high FGF9 treatment compared with the control (*P* < 0.05). Data represent the mean ±SEM.

It was previously shown that FGF9 stimulates proliferation of precursors of Sertoli cells in developing male gonads [31]. Undifferentiated PGCs continuously proliferate until they enter either meiosis (female pathway) or mitotic arrest (male pathway) [32]. Because our data showed that high levels of FGF9 did not induce either male or female differentiation of PGCs (Figures 1A–C and 2), we suspected that high levels of FGF9 might stimulate PGC proliferation in vitro. To test this hypothesis, we cultured 12.5 dpc XY PGCs in the presence of low (0.2 ng/ml) or high (25 ng/ml) concentration of FGF9 for 2 days and then subjected the cells to an EdU cell proliferation assay (Figure 3). In PGCs cultured in the presence of low FGF9, only 10% of the cells became EdU positive and there was no significant difference relative to the control cells which were 5% EdU positive (*P* > 0.05) (Figure 3A and B). In contrast, in the presence of high FGF9, 40% of the PGCs became EdU positive indicating a significantly enhanced level of proliferation relative to the control (*P* < 0.05) (Figure 3A and B), suggesting that DNA replication was stimulated by high FGF9 treatment. These results indicate that proliferation of XY PGCs is selectively induced by high but not low FGF9 treatment. Taken together, our data strongly support the hypothesis that FGF9 levels regulate the fate of XY PGCs in a dose-dependent manner.

### Signal transduction pathways activated by low and high levels of fibroblast growth factor 9

**Figure 4.**
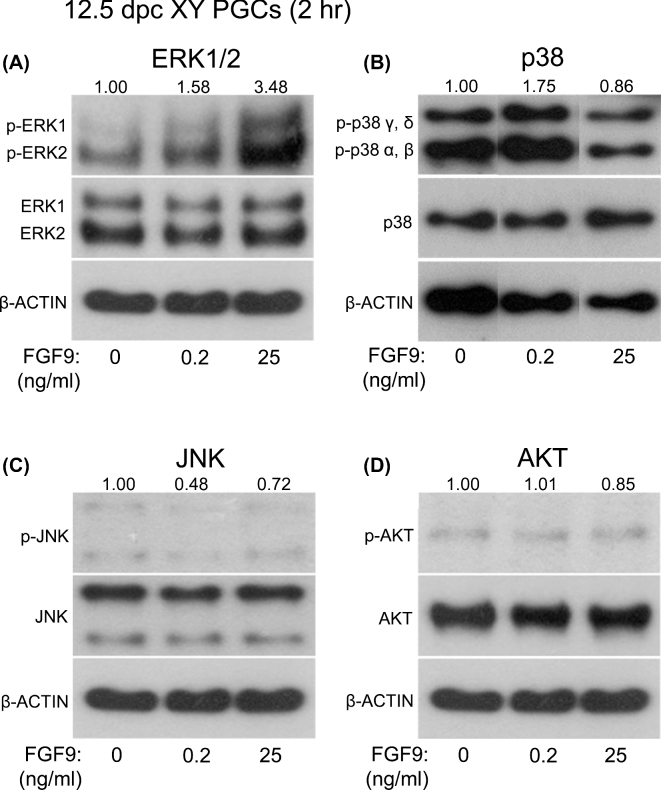
MAPK and AKT signal transduction pathways in XY PGCs cultured with low and high FGF9. To determine whether FGF9 regulates activities of MAPK (ERK1/2, p38, JNK) and AKT signaling pathways, 10,000 XY PGCs were isolated from 12.5 dpc male gonads and incubated with low and high FGF9 for 2 hr, then subjected to western blot analysis. ERK1/2 activity was markedly enhanced in the cells cultured with high FGF9 (A). In contrast, p38 phosphorylation was promoted in the cells treated with low FGF9 (B). JNK and AKT signaling pathways were not enhanced in XY PGCs following either low or high FGF9 treatment (C, D). The levels of protein bands were quantified by densitometry and normalized to the β-ACTIN levels. The relative optical densities of phosphorylated protein levels were calculated based on the control levels set as 1.00.

The results described above suggest that low FGF9 treatment induces male differentiation of XY PGCs, while high FGF9 treatment stimulates proliferation of these PGCs (Figures 1–3). Therefore, we hypothesized that these different effects of FGF9 might be regulated via the function of different signal transduction pathways. It has been reported that growth factors stimulate MAPK and AKT signaling pathways to induce cellular differentiation and proliferation [33–35] and that the p38 signaling pathway is activated in XY PGCs prior to sex differentiation [25]. Using western blotting, we examined whether low (0.2 ng/ml) or high (25 ng/ml) level of FGF9 promotes phosphorylation of MAPK (ERK1/2, p38, JNK) and AKT signaling pathways in isolated 12.5 dpc XY PGCs (Figure 4). ERK1/2 phosphorylation was predominantly enhanced in the presence of high FGF9 for 2 h, and ERK1/2 activity was elevated to about 3.5-fold higher than that of the control (Figure 4A). On the other hand, low FGF9 treatment led to only a slight activation of the ERK1/2 signaling pathway (1.6-fold of the control level) (Figure 4A). Low FGF9 promoted p38 activity about 1.8-fold of the control level, while high level of FGF9 did not change p38 activity in XY PGCs (∼0.9-fold of the control level) (Figure 4B and Supplemental Figure S1). In contrast, JNK phosphorylation was decreased in the presence of either low or high FGF9 (Figure 4C and Supplemental Figure S2). The AKT signaling pathway showed no changes in activity in PGCs cultured in the presence of either low or high FGF9 (Figure 4D and Supplemental Figure S2). These results suggest that different levels of FGF9 promote different signal transduction pathways in XY PGCs to regulate either proliferation or male differentiation of these cells.

### The effect of the ERK1/2 signaling pathway on primordial germ cell proliferation

**Figure 5.**
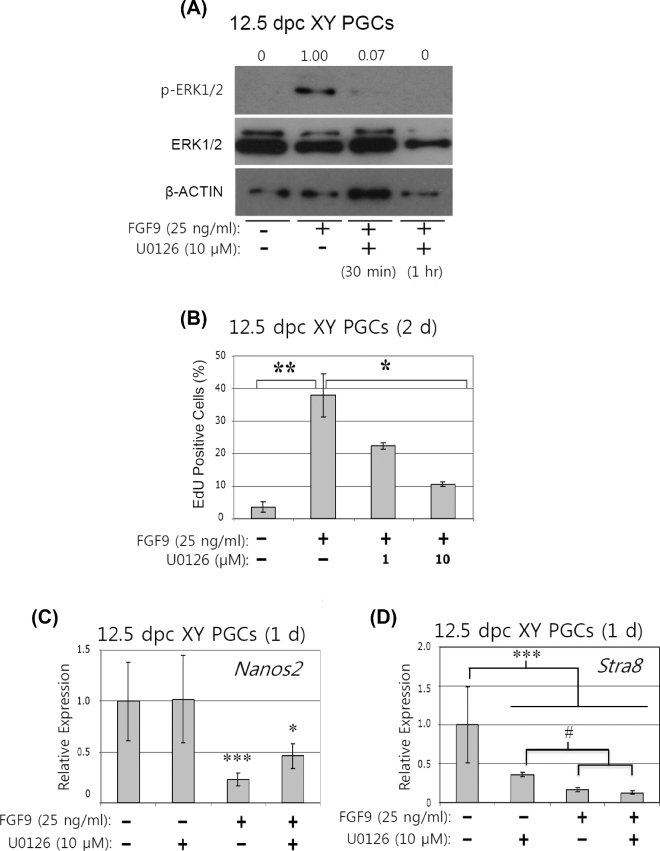
The effect of ERK1/2 signaling activity on proliferation of XY PGCs cultured with high FGF9. **A**) Five thousand XY PGCs isolated from 12.5 dpc male gonads were incubated with high FGF9 for 2 hr to promote phosphorylation of ERK1/2. When the cells were incubated with both high FGF9 and U0126 MEK inhibitor (10 μM) for 30 min or 1 hr, ERK1/2 activity induced by high FGF9 was completely suppressed. The levels of protein bands were quantified by densitometry and normalized to the β-ACTIN levels. The relative optical densities of phosphorylated protein levels were calculated based on the level in the high FGF9 treatment group set as 1.00. **B)** To determine the relationship between the ERK1/2 signaling pathway and PGC proliferation when stimulated by high FGF9 treatment, XY PGCs isolated at 12.5 dpc were cultured with high FGF9 and U0126 inhibitor (1, 10 μM) for 2 days and then subjected to the EdU cell proliferation assay. Cells treated with high FGF9 alone significantly increased cell proliferation (*P* < 0.01). In the presence of high FGF9 and U0126, the ratio of EdU positive cells was clearly diminished in a dose-dependent manner (*P* < 0.05). Data represent the mean ±SEM. ^*^P < 0.05; ^*^^*^P < 0.01. **C, D)** XY PGCs isolated at 12.5 dpc were cultured with or without high FGF9 and/or 10 μM U0126 for 1 day and mRNA levels of *Nanos2* (C) and *Stra8* (D) was examined. In the cells cultured with U0126, *Nanos2* mRNA level was the same as in control cells (*P* > 0.05). In the presence of high FGF9, *Nanos2* expression was significantly suppressed in XY PGCs (*P* < 0.001). In the presence of high FGF9 and U0126, *Nanos2* mRNA expression recovered to a level two times higher than that following culture with high FGF9 treatment alone. In contrast, *Stra8* mRNA levels were significantly low following culture with either high FGF9 alone or high FGF9 and U0126 condition compared with the control (*P* < 0.001) and U0126 alone (*P* < 0.05). Results were normalized to the β-actin mRNA expression. All expression values were calculated relative to control levels set at 1.0. Data represent the mean ±SEM. ^*^P < 0.05 vs. Control; ^*^^*^^*^P < 0.001 vs. Control; #*P* < 0.05 vs. U0126.

We next investigated whether the ERK1/2 signaling pathway activated by high FGF9 treatment regulates PGC proliferation. For this purpose, we used the MEK inhibitor U0126. This compound inhibits the activation of ERK1/2 by inhibiting the kinase activity of MAP kinase kinase (MAPKK or MEK). First, we tested whether U0126 suppresses the phosphorylation of ERK1/2 in XY PGCs in the presence high FGF9 (Figure 5A). High FGF9 treatment for 2 h clearly promoted phosphorylation of ERK1/2 in XY PGCs (Figure 5A). However, this phosphorylation activity was almost completely suppressed in the presence of 10 μM U0126 for 30 min or 1 h (Figure 5A), confirming the efficacy of this inhibitor in cultured XY PGCs.

Using the MEK inhibitor U0126, we then investigated whether or not PGC proliferation induced by high FGF9 treatment is directly regulated by the ERK 1/2 signaling pathway. XY PGCs at 12.5 dpc were cultured in the presence of high FGF9 for 2 days with or without the addition of MEK inhibitor followed by the assessment of proliferation activity based on the EdU assay (Figure 5B). In the presence of high FGF9 without the MEK inhibitor, about 40% of the cells were EdU positive, which represented a significant increase relative to the control value (*P* < 0.01). With the addition of MEK inhibitor (1 or 10 μM), the fraction of EdU-positive cells was gradually decreased in a dose-dependent manner. In particular, the addition of 10 μM MEK inhibitor statistically suppressed PGC proliferation to a level of only 10% of the cultured cells (*P* < 0.05) (Figure 5B). These results strongly suggest that high level of FGF9 stimulates the ERK 1/2 signaling pathway in XY PGCs to promote their proliferation.

Next, we examined the relationship between the ERK 1/2 signaling pathway and PGC sex differentiation. When XY PGCs at 12.5 dpc were cultured without FGF9 for 1 day, male-specific expression of *Nanos2* mRNA remained unchanged in the presence or absence of MEK inhibitor (Figure 5C). However, the addition of high FGF9 resulted in a significant decrease in *Nanos2* mRNA expression in XY PGCs to 25% of the control level without the inhibitor (*P* < 0.001) (Figure 5C). When MEK inhibitor was included with the high level of FGF9, *Nanos2* expression recovered and became 2-fold higher than that detected in PGCs cultured with high FGF9 alone (Figure 5C). We also analyzed female-specific *Stra8* mRNA expression in the presence of high FGF9 and/or MEK inhibitor (Figure 5D). *Stra8* expression was significantly decreased in XY PGCs in the presence of high FGF9 or MEK inhibitor after 1 day of culture (*P* < 0.001). With the addition of both high FGF9 and MEK inhibitor, *Stra8* mRNA expression was continuously suppressed in the cells (*P* < 0.001). Taken together, these results indicate that the ERK1/2 signaling pathway is stimulated by high FGF9 and this induces PGC proliferation, resulting in the suppression of PGCs to enter mitotic arrest (G0 stage), an essential process for PGC male differentiation.

### Inhibition of the p38 signaling pathway activated by low fibroblast growth factor 9

**Figure 6.**
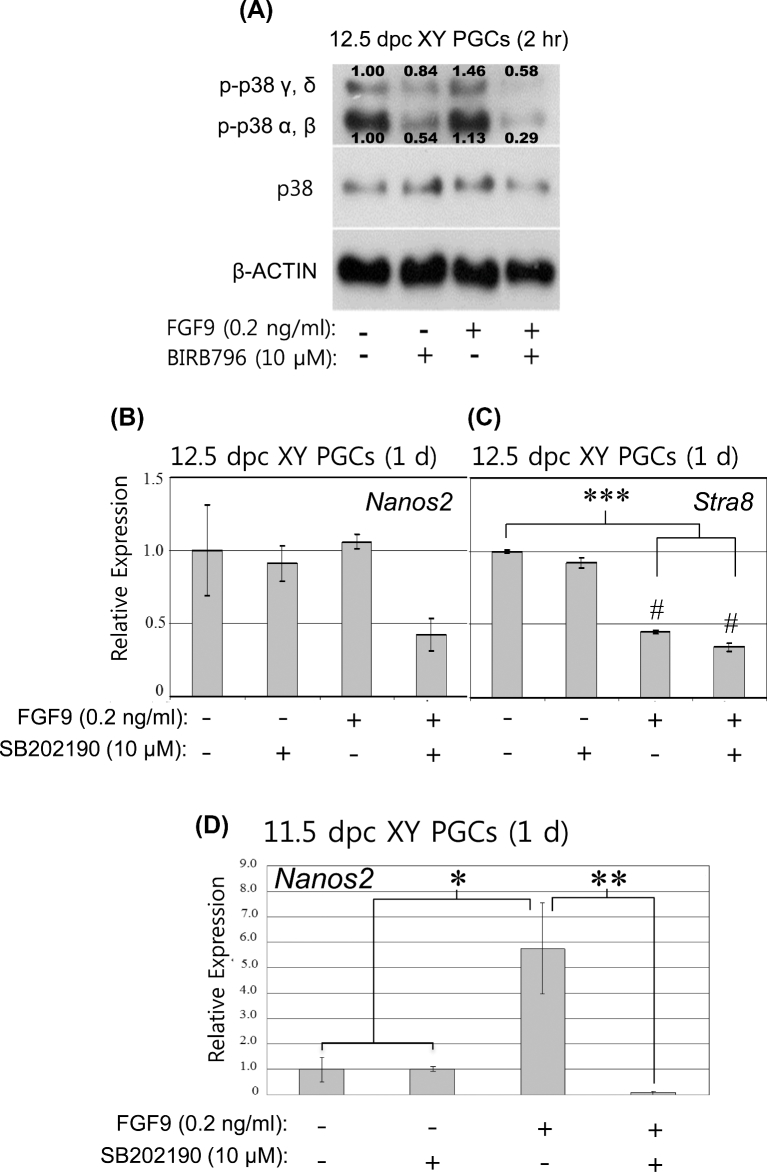
The effect of p38 signaling activity on male differentiation in XY PGCs cultured with low FGF9. **A**) To determine the effect of low FGF9 treatment on phosphorylation of four p38 isoforms (α, β, γ, δ), 10,000 XY PGCs isolated from 12.5 dpc male gonads were cultured with low FGF9 with or without 10 μM BIRB796, an inhibitor of all four p38 isoforms, for 2 hr. BIRB796 treatment moderately suppressed phosphorylation of all isoforms. In the presence of low FGF9 alone, phosphorylation of 4 isoforms was elevated in the cells. In the presence of low FGF9 and BIRB796, activities of all isoforms was suppressed, particularly those of the p38 α and β isoforms which were severely diminished. The levels of protein bands were quantified by densitometry and normalized to the β-ACTIN levels. The relative optical densities of phosphorylated protein levels (the p38 α and β isoforms, the p38 γ and δ isoforms) were calculated based on the control levels set as 1.00. **B, C)** To confirm the relationship between the p38 signaling pathway and PGC male differentiation, XY PGCs isolated at 12.5 dpc were cultured with low FGF9 and/or 10 μM SB202190 inhibitor, which predominantly suppresses p38 α and β isoforms, and mRNA expression levels of *Nanos2* (B) and *Stra8* (C) were examined. After 1 day of culture with low FGF9 alone, *Nanos2* expression was only slightly increased in the cells (*P* > 0.05), but *Stra8* expression was decreased to less than half of the control level (*P* < 0.001). In the presence of both low FGF9 and SB202190, the mRNA level of *Nanos2* expression was less than half of that seen following low FGF9 treatment (*P* > 0.05), although *Stra8* mRNA expression was not increased (*P* < 0.001). Results were normalized to the β-actin mRNA expression. All expression values were calculated relative to control levels set at 1.0. Data represent the mean ±SEM. ^*^^*^^*^P < 0.001 vs. Control; #*P* < 0.05 vs. SB202190. **D)** To determine the relationship between the p38 signaling pathway and male differentiation in sexually indifferent PGCs, XY PGCs at 11.5 dpc were cultured with low FGF9 and/or 10 μM SB202190 inhibitor and *Nanos2* mRNA expression was examined. Low FGF9 treatment significantly enhanced *Nanos2* mRNA expression in the cells after 1 day of culture (*P* < 0.05). In the presence of both low FGF9 and SB202190 inhibitor, *Nanos2* expression was completely suppressed in XY PGCs (*P* < 0.01). Data represent the mean ±SEM. Results were normalized to the β-actin mRNA expression. All expression values were calculated relative to the control level set at 1.0. ^*^P < 0.05; ^*^^*^P < 0.01.

Because low FGF9 treatment enhanced activity of the p38 signaling pathway in XY PGCs (Figure 4B), we next examined whether or not the p38 signaling pathway directly regulates PGC male differentiation. It is known that p38 consists of four isoforms (alpha, beta, gamma, delta) [25]. BIRB796, a p38 inhibitor, binds to the conserved DFG motif to inhibit both the phosphorylation and catalytic activities of all four isoforms (alpha, beta, gamma, delta). To determine the effect of low FGF9 treatment on activities of p38 isoforms, 12.5 dpc XY PGCs were incubated with or without the inhibitor and/or low FGF9 for 2 h (Figure 6A). BIRB796 treatment decreased the phosphorylation activity of all four isoforms of p38 in XY PGCs, suppressing phosphorylation of alpha and beta isoforms more severely than that of gamma and delta isoforms in XY PGCs (0.54- and 0.84-fold of the control, respectively) (Figure 6A). In the presence of low FGF9, phosphorylation of both alpha and beta and gamma and delta isoforms was upregulated (1.13- and 1.46-fold of the control, respectively) (Figure 6A). However, activities of p38 isoforms induced by low FGF9 treatment were significantly suppressed by the addition of BIRB796 inhibitor (Figure 6A). In particular, phosphorylation of the alpha and beta isoforms was severely diminished compared to that of the gamma and delta isoforms (0.29- and 0.58-fold of the control, respectively) (Figure 6A). These results show that the inhibitor successfully suppresses the p38 signaling pathway in XY PGCs that is otherwise stimulated in the presence of low FGF9.

### The effect of inhibition of p38 α and β isoforms on primordial germ cell male differentiation

Finally, we assessed whether or not the p38 signaling pathway promoted by low FGF9 treatment directly regulates PGC male differentiation. In particular, we focused on the role of the alpha and beta of p38 isoforms during PGC male differentiation. We used SB202190, which specifically inactivates the alpha and beta isoforms of p38 by selectively binding to the ATP-binding site of these isoforms to inhibit catalytic activity. We then examined the relationship between p38 signaling and PGC male differentiation in 12.5 dpc XY PGCs (Figure 6B and C). In the cells treated with the SB202190 inhibitor for 1 day, *Nanos2* mRNA expression was slightly diminished compared with the control (*P* > 0.05) (Figure 6B). In this experiment, low FGF9 treatment for 1 day did not sufficiently promote *Nanos2* mRNA expression compared with the control (*P* > 0.05) (Figure 6B), but female-specific *Stra8* mRNA level was reduced to less than half of the control (*P* < 0.001) (Figure 6C). Under both low FGF9 and the inhibitor treatment, *Nanos2* expression level was decreased to less than half of the control (*P* > 0.05) (Figure 6B), although *Stra8* mRNA level was still very low (*P* < 0.001) (Figure 6C). These data indicate that the p38 signal pathway stimulated by low FGF9 treatment is critical to promote PGC male differentiation.

We also analyzed *Nanos2* mRNA expression in sexually indifferent XY PGCs at 11.5 dpc in the presence or absence of low FGF9 and/or SB202190 for 1 day (Figure 6D). The inhibitor treatment without the addition of exogenous FGF9 did not affect *Nanos2* expression in the cells. In the presence of low FGF9, *Nanos2* expression was significantly enhanced to a level nearly 6-fold higher than that of the control (*P* < 0.05) (Figure 6D), confirming our previous result (Figure 2). However, stimulation of *Nanos2* mRNA expression promoted by low FGF9 treatment was completely inhibited by the simultaneous addition of SB202190 (*P* < 0.01) (Figure 6D). Although we did not determine the roles of the gamma and delta of p38 isoforms during PGC male differentiation, our present data indicate that the alpha and beta isoforms of p38 signaling pathway that have been stimulated by low FGF9 treatment play a key role in promoting PGC male differentiation.

## Discussion

It was reported that FGF9 acts directly on PGCs to induce male differentiation [17,20], but it is not clear which specific mechanisms are subject to FGF9 regulation. In this study, we have demonstrated that FGF9 can directly regulate two different fates of XY PGCs in a dose-dependent manner: (1) a low level of FGF9 (0.2 ng/ml) induces PGCs to enter the male differentiation pathway and (2) a high level of FGF9 (25 ng/ml) promotes proliferation of PGCs while suppressing their differentiation. We also found that low and high levels of FGF9 stimulate different MAPK signal transduction pathways in XY PGCs. Thus, low FGF9 treatment selectively activates the p38 MAPK pathway in XY PGCs to promote entry of PGCs into the male differentiation pathway, while high FGF9 treatment induces upregulation of the ERK1/2 pathway in XY PGCs which triggers PGC proliferation. Thus, it appears that distinct MAPK signaling pathways, each stimulated by FGF9 in a dose-dependent manner, regulate a balance between XY PGC proliferation and entry into the male differentiation pathway.

FGF family members are known to be involved in both cell proliferation and cell differentiation processes [36,37]. Our observation that low FGF9 treatment induces male differentiation of XY PGCs is consistent with previous reports that FGF9 functions as a factor that directs PGCs into the male differentiation pathway [17,20]. Similarly, our observation that high FGF9 treatment stimulates mitotic proliferation of PGCs resulting in the inhibition of PGC differentiation is consistent with previous reports that FGF family members, including bFGF, FGF4, and FGF8, stimulate mouse PGC proliferation in vitro [38–40], as well as proliferation of postnatal spermatogonia in culture [41]. After PGCs migrate into the XY gonad by 11.5 dpc, the cells continue mitotic cell division until 13.5 dpc or later, when gonadal XY PGCs cease proliferation and enter mitotic arrest (G0) [2]. Importantly, the transition from mitotic proliferation to mitotic arrest in XY PGCs coincides with stage-specific, dynamic changes in expression of FGF9 in the murine XY gonad, progressing from extremely high levels in the 11.5 dpc gonad to decreased levels by 12.5–14.5 dpc [15–17]. Although we do not know whether the doses of FGF9 we set here (high: 25 ng/ml, low: 0.2 ng/ml) are comparable to the physiological levels of FGF9 in the developing male gonad, these previous studies plus our current results strongly suggest that FGF9 produced by Sertoli cells developing in the XY gonad functions as a direct regulator of male (XY) PGC fate in a stage-specific, dose-dependent manner.

To investigate the mechanisms of PGC male differentiation, we previously cultured isolated XX and XY PGCs at 11.5 and 12.5 dpc with RA receptor inhibitor. Although *Stra8* mRNA expression was suppressed in all of the groups, *Dnmt3l* mRNA expression was elevated only in 12.5 dpc XY PGCs [29]. These data suggest that inhibiting RA signaling is not sufficient for PGC male differentiation but the factor(s) in male gonadal environment also contribute to this pathway. Interestingly, only XY PGCs, but not XX PGCs, responded to different levels of FGF9 in this study. Our data show the XY-specific response to FGF9 occurs even in PGCs isolated from sexually undifferentiated gonads at 11.5 dpc. It has been reported that XX and XY PGCs express the same FGF receptors, FGFR1, FGFR3, and FGFR4 [18], suggesting the XY-specific PGC response to FGF9 is not dependent on the bias of a specific ligand–receptor combination. DiNapoli et al. demonstrated that FGF9 functions as an XY-specific survival factor in the early-stage mouse gonad [19]. In XY *Fgf9*-deficient gonads, most of the PGCs were lost between 11.5 and 12.5 dpc, whereas disruption of *Fgf9* had no effect on survival of PGCs in XX gonads. Because XX PGCs isolated from the testes of *Sry*^Myc^ transgenic embryos [13] respond to FGF9 treatment in vitro, it has been suggested that the XY-specific response to FGF9 is conferred by the XY gonadal environment, rather than by the XY chromosomal constitution of the PGCs [19]. However, PGCs may synthesize sex-specific cofactors such as heparan sulfate proteoglycans [19]. Actually, it is known that heparan molecules bind to both FGFRs and FGF ligands on the cell surface, and contribute to stabilization of the FGFR-FGF ligand complex (reviewed by [37,42]). Therefore, it has been suggested that the XY gonad at 11.5 dpc already synthesizes sex-specific factors such as heparan sulfate molecules, which predominantly support the FGFR-FGF9 complex, causing a sex-specific response to FGF9 in XY PGCs only.

Our current data show that XY PGCs exposed to different concentrations of FGF9 (low versus high) upregulate different signal transduction pathways (p38 versus ERK1/2) in XY PGCs, resulting in distinct cellular responses. Previously, it has been reported that a single cell type can exhibit divergent responses to the same FGF dependent on the exposure concentration, and that this is controlled by distinct signaling pathways. Garcia-Maya et al. reported that 3T3 fibroblasts treated with FGF2 at different concentrations provide pleiotropic cellular responses: proliferation, survival, and differentiation [43]. In that study, FGF2 at low concentrations (<1 ng/ml) stimulated cells to survive and differentiate, while intermediate concentrations (1–10 ng/ml) induced cells to proliferate, and high concentrations (100 ng/ml) reversed the effects of intermediate concentrations by inhibiting proliferation and stimulating cell survival. They further demonstrated that FGF2 concentration precisely controls activities of the ERK and p38 MAPK signaling pathways in cultured cells [43].

It has been also reported that FGF family members regulate two different fates of lens epithelial cells in a dose-dependent manner: cell proliferation at a low concentration and differentiation at a high concentration [44]. Lovicu and McAvoy demonstrated that dose-dependent functions of FGF2 in cells are regulated by the dose-dependent activation levels of the ERK1/2 pathway [45]. It has also been suggested that the duration of ERK activity is critical to the regulation of cell fates [46]. A high concentration of FGF maintained a prolonged ERK1/2 activity, whereas a lower concentration of FGF shortened the activation period of ERK1/2 signaling, inducing cell differentiation or proliferation, respectively [47]. It is not fully understood how these dose-dependent mechanisms function, but it is suggested that competition between binding of the FGF ligand to heparan sulfate glycosaminoglycans and FGF receptors leads to different responses in cells [48]. Nevertheless, it is clear that different FGF concentrations can differentially regulate intracellular signaling and, thus, cellular responses to this growth factor, and that this has important implications for understanding how quantitative and temporal changes in FGF concentrations can influence cell proliferation, migration, survival, and differentiation during embryo development, including the regulation of XY PGC proliferation and/or differentiation.

**Figure 7.**
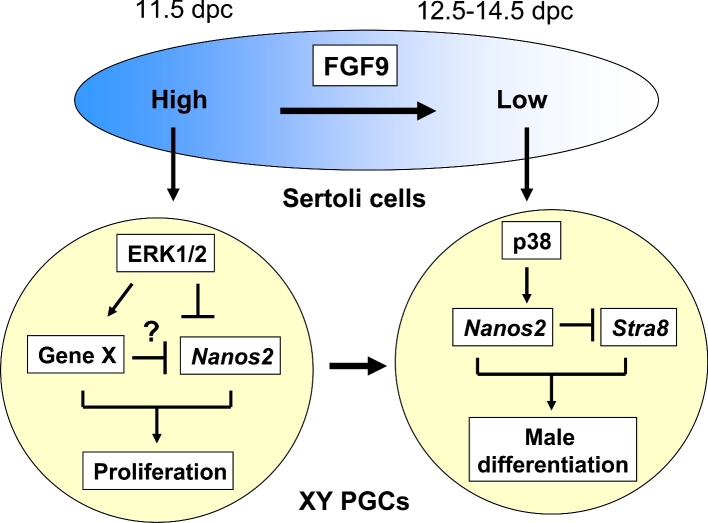
Model for the fate decision of XY PGCs regulated by stage-specific FGF9 levels. Before sex differentiation at 11.5 dpc, FGF9 expression is upregulated in undifferentiated Sertoli cells. At 12.5-14.5 dpc, FGF9 levels are gradually decreased while testis differentiation progresses. The stage-specific expression of FGF9 in XY gonads regulates the different signaling transduction pathways in XY PGCs. High FGF9 stimulates the ERK1/2 signaling pathway to promote unknown genes and suppress *Nanos2* expression, resulting in PGC proliferation. In contrast, low FGF9 enhances the p38 signaling pathway to induce *Nanos2* expression for PGC male differentiation.

Recently several studies have reported that the TGFβ superfamily Nodal/Activin signaling also regulates PGC male differentiation [21–24,26]. Spiller et al. found that *Nodal* and *Cripto*, a Nodal coreceptor, are predominantly expressed in XY germ cells during mouse gonadal development [26]. Following FGF9 treatment, *Cripto*, but not *Nodal* expression, was enhanced in cultured PGCs [26], suggesting that FGF9 signaling does not regulate Nodal activity for PGC differentiation. Wu et al. also demonstrated that Nodal/Activin signaling contributes to PGC male differentiation independent of FGF9 signaling [24]. Our study demonstrated that FGF9 signaling induces PGC male differentiation through the p38 MAPK activity. Actually, male germ cell-specific activation of the p38 MAPK pathway was observed in mouse gonads around the time of sex differentiation [25]. In the presence of p38 inhibitors, PGCs in the XY gonad enhance the expression of meiotic marker genes to promote entry into meiosis [25]. Wu et al. further reported that the p38 MAPK signaling dose not induce *Nanos2* expression, but suppresses RA signaling in XY PGCs [23]. Instead, SMAD2 stimulated by Nodal/Activin signaling actually promotes *Nanos2* expression in male germ cells. They concluded that SMAD2 and p38 signaling act cooperatively to induce *Nanos2* expression and suppress RA signaling in male germ cells [23]. In contrast, our present results suggest that suppression of the p38 pathway does not promote the meiotic process in isolated XY PGCs. This difference in results might be dependent on culture conditions—specifically culturing PGCs with or without a gonadal somatic environment. We previously determined that meiotic inhibition itself is necessary but not sufficient to induce PGC male differentiation [29]. Our isolated PGC culture system has now allowed us to determine the direct effect of p38 MAPK signaling pathway on PGC male differentiation. Thus, our results suggest that FGF9 produced by Sertoli cells is a key regulator of PGC differentiation in the XY gonad before and after sex differentiation. In conclusion, our in vitro study proposes that the stage-specific expression of FGF9 in XY gonads regulates different signal transduction pathways, ERK1/2 and p38, in XY PGCs, resulting in the balance between proliferation and differentiation of the cells in a dose-dependent manner (Figure 7).

## Supplementary data

Supplementary data are available at *BIOLRE* online.

**Supplemental Table S1.** Antibody information used for western blotting.

**Supplemental Figure S1.** Original western blot image of Figure 4B. To determine the effect of FGF9 on the p38 signaling activity, 10,000 XY PGCs isolated from 12.5 dpc male gonads were cultured with low or high FGF9 in the presence or absence of factor X for 2 h to be subjected to western blot analysis. The images of lane 1 (control), lane 3 (low FGF9 alone), and lane 5 (high FGF9 alone) were trimmed and arranged to sit next each other to provide Figure 4B.

**Supplemental Figure S2.** The activities of primary antibodies against p-AKT and p-JNK. To ensure the binding affinity of antibodies of p-AKT (1:500) and p-JNK (1:500), freshly collected proteins obtained from adult testis and 12.5–15.5 dpc male gonads were subjected to western blot analysis under the same condition in Figure 4C and D. Equal amount of total protein (28 μg/well) were loaded into the well on 12% SDS-PAGE gel. The p-AKT antibody specifically recognized the AKT signaling activities in all samples tested. The p-JNK antibody also successfully recognized the phosphorylated form of JNK in adult testis and 15.5 dpc male gonads. These preliminary results have demonstrated that p-AKT and p-JNK primary antibodies we used are reliable to recognize those active forms.

Supplemental material Supplementary data are available at *BIOLRE* online.
**Supplemental Table S1.** Antibody information used for western blotting.
**Supplemental Figure S1.** Original western blot image of Figure 4B. To determine the effect of FGF9 on the p38 signaling activity, 10,000 XY PGCs isolated from 12.5 dpc male gonads were cultured with low or high FGF9 in the presence or absence of factor X for 2 h to be subjected to western blot analysis. The images of lane 1 (control), lane 3 (low FGF9 alone), and lane 5 (high FGF9 alone) were trimmed and arranged to sit next each other to provide Figure 4B.
**Supplemental Figure S2.** The activities of primary antibodies against p-AKT and p-JNK. To ensure the binding affinity of antibodies of p-AKT (1:500) and p-JNK (1:500), freshly collected proteins obtained from adult testis and 12.5–15.5 dpc male gonads were subjected to western blot analysis under the same condition in Figure 4C and D. Equal amount of total protein (28 μg/well) were loaded into the well on 12% SDS-PAGE gel. The p-AKT antibody specifically recognized the AKT signaling activities in all samples tested. The p-JNK antibody also successfully recognized the phosphorylated form of JNK in adult testis and 15.5 dpc male gonads. These preliminary results have demonstrated that p-AKT and p-JNK primary antibodies we used are reliable to recognize those active forms.
