# Supplementary figures and images for: Dose-dependent functions of fibroblast growth factor 9 regulate the fate of murine XY primordial germ cells
Source: Biol Reprod. 2016 Dec 23;96(1):122–33. doi: 10.1095/biolreprod.116.143941 (PMC5803787; doi:10.1095/biolreprod.116.143941)

# Supplemental Figure S1

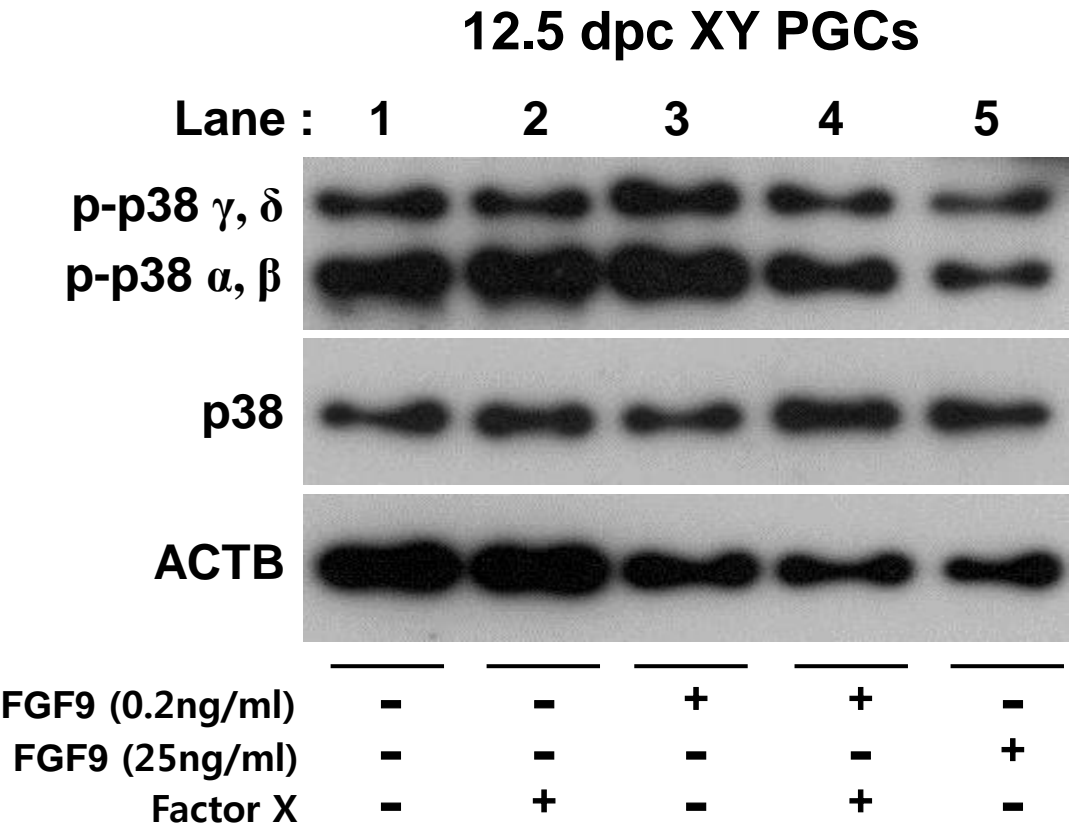

# Supplemental Figure S2

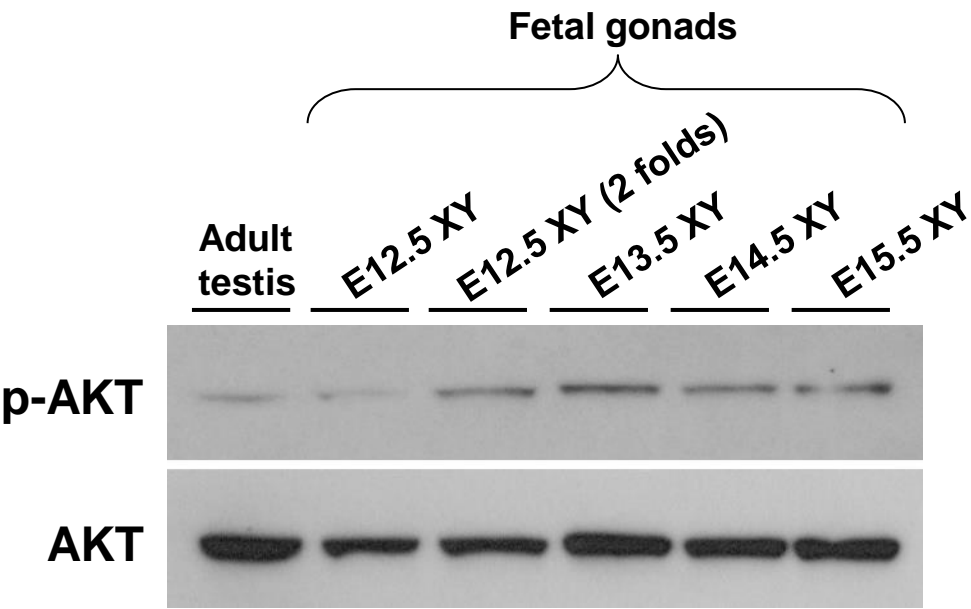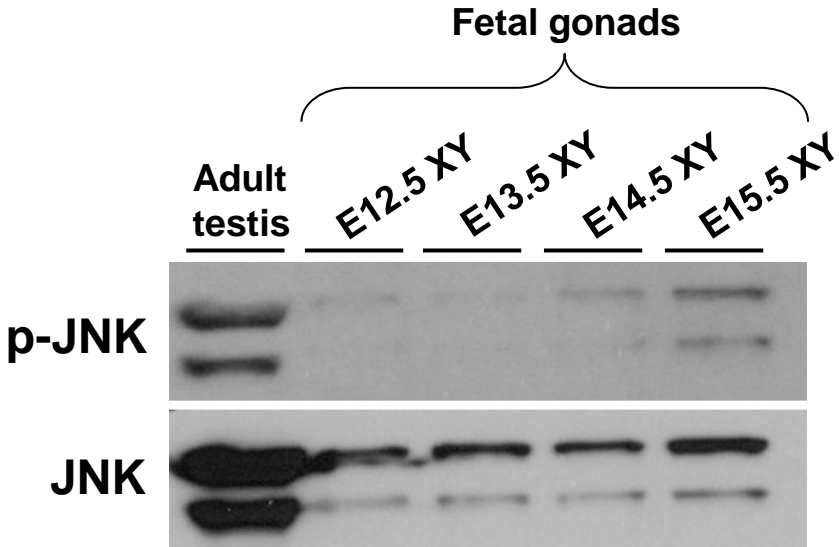

Supplement: Supplemental material — Supplementary data are available at BIOLRE online. Supplemental Table S1. Antibody information used for western blotting. Supplemental Figure S1. Original western blot image of Figure 4B. To determine the effect of FGF9 on the p38 signaling activity, 10,000 XY PGCs isolated from 12.5 dpc male gonads were cultured with low or high FGF9 in the presence or absence of factor X for 2 h to be subjected to western blot analysis. The images of lane 1 (control), lane 3 (low FGF9 alone), and lane 5 (high FGF9 alone) were trimmed and arranged to sit next each other to provide Figure 4B. Supplemental Figure S2. The activities of primary antibodies against p-AKT and p-JNK. To ensure the binding affinity of antibodies of p-AKT (1:500) and p-JNK (1:500), freshly collected proteins obtained from adult testis and 12.5–15.5 dpc male gonads were subjected to western blot analysis under the same condition in Figure 4C and D. Equal amount of total protein (28 μg/well) were loaded into the well on 12% SDS-PAGE gel. The p-AKT antibody specifically recognized the AKT signaling activities in all samples tested. The p-JNK antibody also successfully recognized the phosphorylated form of JNK in adult testis and 15.5 dpc male gonads. These preliminary results have demonstrated that p-AKT and p-JNK primary antibodies we used are reliable to recognize those active forms. [file bio143941_supp.pdf]
